# Inhibition of PERK Kinase, an Orchestrator of the Unfolded Protein Response (UPR), Significantly Reduces Apoptosis and Inflammation of Lung Epithelial Cells Triggered by SARS-CoV-2 ORF3a Protein

**DOI:** 10.3390/biomedicines11061585

**Published:** 2023-05-30

**Authors:** Panagiotis Keramidas, Eleni Papachristou, Rigini M. Papi, Aglaia Mantsou, Theodora Choli-Papadopoulou

**Affiliations:** Laboratory of Biochemistry, Department of Chemistry, Aristotle University of Thessaloniki, University Campus, 54124 Thessaloniki, Greece; pankerdim@chem.auth.gr (P.K.); epapachristou@chem.auth.gr (E.P.); rigini@chem.auth.gr (R.M.P.); mantsouav@chem.auth.gr (A.M.)

**Keywords:** SARS-CoV-2, ORF3a, apoptosis, ER stress, pyroptosis, cytokine storm, inflammation, PERK, GSK2606414

## Abstract

SARS-CoV-2 ORF3a accessory protein was found to be involved in virus release, immunomodulation and exhibited a pro-apoptotic character. In order to unravel a potential ORF3a-induced apoptotic and inflammatory death mechanism, lung epithelial cells (A549) were transfected with in vitro synthesized *ORF3a* mRNA. The protein’s dynamic involvement as “stress factor” for the endoplasmic reticulum, causing the activation of PERK kinase and other UPR-involved proteins and therefore the upregulation of their signaling pathway executioners (ATF6, XBP-1s, PERK, phospho eIF2a, *ATF4*, CHOP, *GADD34*), has been clearly demonstrated. Furthermore, the overexpression of BAX and BH3-only pro-apoptotic protein PUMA, the upregulation of Bcl-2 family genes (*BAX, BAK, BID, BAD*), the reduced expression of Bcl-2 in mRNA and protein levels, and lastly, the cleavage of PARP-1 and caspase family members (caspase-3,-8 and -9) indicate that ORF3a displays its apoptotic character through the mitochondrial pathway of apoptosis. Moreover, the upregulation of *NFκB*, phosphorylation of p65 and IκΒα and the elevated expression of pro-inflammatory cytokines (IL-1b, IL-6, IL-8 and IL-18) in transfected cells with *ORF3a* mRNA indicate that this protein causes the inflammatory response through NFκB activation and therefore triggers lung injury. An intriguing finding of our study is that upon treatment of the ORF3a-transfected cells with GSK2606414, a selective PERK inhibitor, both complications (apoptosis and inflammatory response) were neutralized, and cell survival was favored, whereas treatment of transfected cells with z-VAD (a pan-caspase inhibitor) despite inhibiting cell death, could not ameliorate the inflammatory response of transfected A549 cells. Given the above, we point out that PERK kinase is a “master tactician” and its activation constitutes the main stimulus for the emergence of ORF3a apoptotic and inflammatory nature and therefore could serve as potential target for developing novel therapeutic approaches against COVID-19.

## 1. Introduction

SARS-CoV-2 is a highly contagious virus that was the cause of the coronavirus disease 2019 (COVID-19) pandemic [1]. It is a member of the beta-coronavirus family and has an enveloped, single-stranded, positive-sense RNA that is approximately 30,000 nucleotides in length [2]. The replicase gene occupies two-thirds of the viral genomic region and may encode non-structural viral proteins (NSPs). The remaining 10-kb region preceding the 3’-end encodes structural proteins, such as spike (S), envelope (E), membrane (M) and nucleocapsid (N) proteins. In addition to structural proteins, nine accessory proteins are encoded by the virus’ genome. [3]. In the case of SARS-CoV-2, accessory proteins have received less attention than other proteins in the viral genome, and much of our knowledge about them is based on comparative studies with homologous proteins of previously characterized coronaviruses, such as SARS-CoV and MERS-CoV. These proteins play a significant role in virus–host interactions and the modulation of host immune responses, contributing to coronaviral pathogenicity through various mechanisms [4]. The ORF3a gene encodes a 274-amino acid protein, which is the largest among the SARS-CoV-2 accessory proteins. SARS-CoV-2 ORF3a shares 72 percent amino acid homology with its SARS-CoV counterpart [5]. Although early findings indicate that this protein is linked to virulence, infectivity, and virus release [6,7,8], the exact mechanisms by which it is involved in disease pathogenesis are uncertain [9].

Apoptosis is a regulated form of cell death that involves a series of molecular steps that results in the elimination of unwanted cells from a tissue and plays an important role in physiological development of living organisms and their tissue homeostasis [10]. When physiology of cells is disrupted due to a pathological state, such as viral infections, immunological problems or degenerative diseases, apoptotic pathways are activated [11]. Apoptosis can be either triggered through an “extrinsic pathway”, characterized by the activation of membrane death receptors, or through increased mitochondria outer membrane permeability (MOMP) and cytochrome c release (“intrinsic” or “mitochondrial” pathway) [11,12,13]. Nevertheless, both mechanisms of apoptosis are driven by the cleavage of specific members of cysteinyl aspartate proteases family, known as caspases [12,14,15]. Pyroptosis is an inflammatory form of cell death, which is triggered by danger signals that are recognized by NOD-Like Receptors (NLR) and induce the release of pro-inflammatory factors [16]. Various death and/or death signals can drive the formation of multiprotein complexes called inflammasomes in both immune and non-immune cells and provoke the inflammatory response of cells [11,16]. Extended activation of inflammasomes triggers the maturation and secretion of pro-inflammatory cytokines, such as IL-1b and IL-18, leading to cell death and regulating adaptive and innate immune responses [17,18]. The activation of inflammasomes is observed in COVID-19 patients and considered to be an appealing therapeutic target for amelioration of the virus-induced cytokine storm [19].

The endoplasmic reticulum (ER) is a membranous eukaryotic organelle that controls the synthesis, folding, maturation and trafficking of cellular proteins. However, several physiological and pathological circumstances can disrupt the protein-folding mechanism, resulting in an increased load of unfolded or misfolded proteins within the organelle lumen. This condition results in a form of cellular stress known as “ER stress”. To detect and adapt to ER stress, cells have developed a conserved mechanism called the “Unfolded Protein Response (UPR)” to counteract this effect [20]. Upon ER stress, three interconnected transmembrane signal transducers (PERK, IRE1 and ATF6) are activated to mediate the UPR and subsequent cellular stress response pathways. PERK, a transmembrane protein kinase located in the ER, plays a dual role (pro-apoptotic and pro-survival) depending on the level of stress applied to cells [21]. Upon stress signaling, PERK is activated by autophosphorylation, and its kinase domain phosphorylates eukaryotic Initiation Factor-2 (eIF2). The phosphorylated eIF2a inhibits the general mechanism of translation, allowing only specific mRNAs, such as those of activating transcription factor 4 (ATF4) and CCAAT/enhancer binding protein homologous protein (CHOP), to be encoded. Activated ATF4 and CHOP result in cellular damage, cell death and inflammatory responses [22]. Concluding, UPR controls various signaling pathways that can affect antiviral response of the host cells and can be used as a target for developing new therapeutic approaches [23].

In the scientific epilogue of our study, we attribute PERK kinase the role of “master tactician” for the direct emergence of ORF3a apoptotic and inflammatory nature. In particular, both apoptosis and inflammation are drastically inhibited by treating the cells with GSK2606414, a selective PERK inhibitor. However, treatment of the transfected cells with z-VAD, a pan-caspase inhibitor, counteracts apoptotic cell death but not NFκB activation, expression and secretion of pro-inflammatory mediators. These results strongly correlate ORF3a-induced apoptotic cell death and inflammatory response with PERK kinase activation and “recommend” PERK kinase inhibitors as potential novel therapeutic approaches against COVID-19.

## 2. Materials and Methods

### 2.1. Cell Lines

Human lung carcinoma A549 cell line (CCL-185) was obtained from ATCC (American Type Culture Collection, Manassas, VA, USA). Cells were cultured in DMEM containing 4.5 g/L glucose, L-glutamine, pyruvate (Fischer, Gibco, Waltham, MA, USA), 10% *v/v* fetal bovine serum (Thermo Fischer, Gibco, Waltham, MA, USA), and 1% *v/v* penicil-lin/streptomycin (Thermo Fischer, Gibco, Waltham, MA, USA). The cells were maintained at 37 °C in a humidified 5% CO_2_ atmosphere.

### 2.2. Plasmid Vectors for In Vitro Transcription and Subcellular Localization of ORF3a

The coding sequence of the SARS-CoV-2 ORF3a protein was cloned into the pT7-IRES-His-C DNA vector (Takara Bio Inc., Kusatsu, Shiga, Japan) using gene-specific oligonucleotide primers containing NdeI and XbaI restriction sites to generate *ORF3a* mRNA via an in vitro transcription reaction. The plasmid vector pGBW-m4134093 (Addgene, Watertown, MA, USA) encoding ORF3a was utilized as a template for PCR amplification of the target gene using the KAPA HiFi PCR Kit (KAPA BIOSYSTEMS, Wilmington, MA, USA). Restriction analysis and DNA sequencing (Eurofins Genomics, Ebersberg, Germany GmbH) were used to demonstrate the presence and integrity of gene inserts, respectively. Then, the recombinant pT7-IRES-His-C DNA/ORF3a vector was digested by NotI, downstream of the T7 promoter, and the linearized plasmid was used as a template for *ORF3a* mRNA synthesis by an in vitro transcription reaction, according to the manufacturer’s protocol (New England Biolabs, Ipswich, MA, USA). ORF3a PCR amplification (approximately 840 bp), using pGBW-m4134093 as the DNA template, cloning into the pT7-IRES-His-C DNA vector. Electrophoresis of in vitro synthesized ORF3a-mRNA are shown in Figure S1 in Supplementary Data.

Green fluorescent protein (GFP) mRNA was used as the control. Thus, GFP gene was cloned in pT77 plasmid vector [24] which contains a T7 promoter. The recombinant pT77 –GFP vector was linearized by restriction enzyme HindIII (Takara Bio Inc., Kusatsu, Shiga, Japan), downstream to T7 promoter and the linearized plasmid was used as template for GFP mRNA synthesis by in vitro transcription reaction with HiScribe T7 ARCA mRNA Kit, according to manufacturer’s protocol (New England Biolabs, Ipswich, MA, USA). Poly-A tailing of ORF3a’s and GFP’s mRNA was carried out using *E. coli* Poly(A) Polymerase (NEB# M0276, New England Biolabs, Ipswich, MA, USA). Synthesized mRNAs were purified using a spin column-based method (Monarch RNA Cleanup Kits, New England Biolabs, Ipswich, MA, USA). To examine the subcellular distribution of the ORF3a protein, the target gene was cloned into the eukaryotic vector pEGFP-N1 between BamHI and XhoI restriction sites. All restriction enzymes used in this study were purchased from Takara Bio, Inc (Takara Bio Inc., Kusatsu, Shiga, Japan). The 1 kb and 50 bp DNA Ladders obtained from Nippon Genetics (Nippon Genetics Europe GmbH, Düren, Germany), whereas the 100 bp Ladder was purchased from New England Biolabs. Primers used for cloning ORF3a in pT7-IRES-His-C DNA and pEGFP-N1 vectors were synthesized by Eurofin Genetics and are listed in Table S1.

### 2.3. Plasmid and mRNA Transfection

A549 cells were plated into a 24-well plate at a density of 2 × 10^4^ cells per well, transfected with 1 μg of the indicated plasmid using Xfect Transfection Reagent (Takara Bio Inc., Kusatsu, Shiga, Japan) and cultured for 48 h.

For transient transfection with ORF3a and GFP mRNAs, A549 cells were plated into a 24-well plate with a concentration of 5 × 10^4^ cells per well and were transfected with 0.5 μg of each mRNA, respectively, using jetMESSENGER^®^ Polyplus SA (Polyplus, Illkirch-Graffenstaden, France), according to manufacturer’s protocol. After cell lysis at 48, 72 and 96 h post-transfection (hpt) RNA and protein extracts were collected for the control group (non-transfected cells), ORF3a group (transfected with ORF3a-mRNA) and GFP group (cells transfected with GFP mRNA).

### 2.4. RNA Extraction and Quantitative RT-PCR

Total RNA was isolated from control and transfected cells using the NucleoSpin RNA kit (Macherey Nagel, Düren, Germany). cDNA was synthesized using the PrimeScript RT Reagent Kit-Perfect Real Time (Takara Bio Inc., Kusatsu, Shiga, Japan). Real-time quantitative PCR (RT-qPCR) assays were performed using the KAPA SYBR^®^ FAST qPCR Kit Master Mix (2X) ABI PRISM (KAPA BIOSYSTEMS, Wilmington, MA, USA) in an Applied Biosystems 96 plate system. The annealing temperature for all primer pairs was set to 60 °C. The qPCR program consisted of the following stages: an initial denaturation stage at 95 °C for 20 s, followed by 40 cycles of amplification (denaturation at 95 °C for 3 s, annealing and extension at 60 °C for 20 s) and, finally, a melt curve stage for each PCR product. All reactions were performed in triplicates. Relative expression of different gene transcripts was calculated by the ΔΔCt method. Data were normalized to *GAPDH* levels. The primers used for the RT-PCR analysis are listed in Table S2 in Supplementary Data [25,26,27,28,29,30,31,32,33].

### 2.5. Western Blotting

Cells were lysed using RIPA Lysis Buffer (Thermo Fisher Scientific, Waltham, MA, USA) and a protease inhibitor cocktail (Thermo Fisher Scientific, Waltham, MA, USA). The total protein concentration was calculated using the Bradford Assay. Equal amounts of total protein (50 µg) per well were separated by SDS-PAGE and transferred to nitrocellulose membrane (Macherey Nagel, Düren, Germany). Membranes were blocked with 5% *w/v* non-Fat Skimmed Milk in PBS for an hour.

The membranes were incubated overnight at 4 °C with the primary antibodies shown below: polyclonal rabbit His-tag, polyclonal rabbit GFP, polyclonal rabbit caspase-3, polyclonal rabbit caspase-8, polyclonal rabbit caspase-9, monoclonal rabbit p-eIF2a, monoclonal rabbit IL-1b, monoclonal rabbit IL-18, monoclonal rabbit IL-6, monoclonal rabbit IL-8, monoclonal mouse CHOP, monoclonal rabbit PUMA, monoclonal rabbit Bcl-2, monoclonal rabbit BAX, monoclonal rabbit XBP-1s, monoclonal rabbit ATF6, polyclonal rabbit PARP1, polyclonal rabbit phospho p65 and monoclonal rabbit phospho IκBα. All were obtained from Cell Signaling Technology. PERK polyclonal rabbit primary antibody was obtained from OriGene (OriGene, Rockville, MD, USA). Polyclonal rabbit GAPDH was purchased from Santa Cruz (sc-25778, Santa Cruz Biotechnology, Inc., Dallas, TX, USA). Appropriate goat anti-mouse (Merck KGaA, Darmstadt, Germany) and anti-rabbit (Cell Signaling Technology, Danvers, MA, USA) IgG AP-conjugated secondary antibodies were incubated with the membrane in 5% *w/v* non-fat skimmed milk for 90 min at room temperature. After washing the membrane with PBS-Tween (0.04% *v/v*), bands were developed in Alkaline Phosphatase Buffer by adding 50 mg/mL BCIP (Biotium, Fremont, CA, USA) and 50 mg/mL NBT (Biotium, Fremont, CA, USA) solutions.

### 2.6. Confocal Microscopy

For immunofluorescence microscopy, 2 × 10^4^ cells were plated on borosilicate glass coverslips in a 24-well plate and transfected either with pEGFP-N1 (control group) or recombinant pEGFP-N1/ORF3a (transfected group) vector, as previously mentioned. Both cell groups were fixed with 4% *w/v* paraformaldehyde and stained with DAPI (0.5 μg/mL, Sigma Aldrich, St. Louis, MO, USA). Images were captured using a confocal microscope at 40× magnification (image scale, 10 μm). Confocal fluorescence microscopy studies were conducted using a Zeiss LSM780 confocal laser scanning microscope (CLSM) (Zeiss, Berlin, Germany), equipped with a Zeiss Axiocam MRc5 digital camera (Zeiss, Berlin, Germany).

### 2.7. Assessment of Cell Viability

The viability of A549 cells treated with PERK inhibitor was assessed by MTT assay. Briefly, cells were seeded in 96-well plates at a density of 5 × 10^3^ cells/well. Twenty-four hours after plating, the cells were treated with the concentrations 250, 500, 1000 and 1250 nM of PERK inhibitor GSK2606414. At 24 and 48 h after treatment, the MTT solution (5 mg/mL in PBS, Thermo Fischer, Gibco, Waltham, MA, USA) was added to each well. After 4 h of incubation at 37 °C, MTT (Thermo Fischer, Invitrogen, Waltham, MA, USA) solution was removed by aspiration. The insoluble formazan crystals were dissolved in DMSO. The optical densities at 570 and 630 nm for each well were determined using a microplate reader (BioTek Plate Reader, Winooski, VT, USA). Relative survival was represented as the absorbance of the treated sample (Abs_570_-Abs_630_, treated)/absorbance of the control group (Abs_570_-Abs_630_, control) × 100%.

### 2.8. Treatment of Cells with Inhibitors

A549 cells transfected with *ORF3a* mRNA were treated at 48 h post-transfection with 1000 nM of the PERK inhibitor GSK2606414 (Merck KGaA, Darmstadt, Germany) previously dissolved in DMSO or pan-caspase inhibitor z-VAD (50 μM, InvivoGen, San Diego, CA, USA). At 96 h post-transfection, cells were lysed, RNA and protein extracts were collected for the control group (non-transfected and untreated cells), ORF3a + GK414 group (cells transfected with *ORF3a* mRNA and treated with GSK2606414), ORF3a group (transfected only with ORF3a-mRNA), GFP group (cells transfected with GFP mRNA) and z-VAD group (cells transfected with *ORF3a* mRNA and treated with z-VAD).

### 2.9. Trypan Blue Dye Exclusion Assay

The viability of A549 transfected cells exposed to *ORF3a* mRNA was investigated by Trypan Blue Dye (Sigma Aldrich, St. Louis, MO, USA) Exclusion Assay according to Thermo Fisher Scientific protocol. In brief, cells were seeded in 24-well cell culture plates and after 22 h of seeding, were transfected with either *ORF3a* mRNA (ORF3a group) or GFP mRNA (GFP group). The time point of transfection was set as time point zero (0 h). There was a group of A549 cells transfected with *ORF3a* mRNA that were also treated 48 h post-transfection with GSK2606414 inhibitor (ORF3a+GSK414 group). At certain time points after transfection (12, 24, 48, 72 and 96 h post-transfection), cells were harvested and centrifuged at 1200 rpm for 10 min. The cell pellet was washed twice with 1× PBS and afterwards was resuspended in 1× PBS. Then, samples were gently mixed in 1:1 proportion with 0.4% trypan blue and incubated for 5 min at room temperature. The ensued samples were loaded in a hemocytometer chamber for cell counting and the results were expressed as % trypan blue positive cells when compared with the control. The kinetics of growth rate of the cells were expressed as the number of the cells against time.

### 2.10. Detection of Secreted Cytokines

For the detection of secreted cytokines, cellular supernatants were collected from each sample group and a protein amount of 1 mg per sample group—as assessed by Bradford assay—was used for TCA precipitation. TCA was added to the supernatants to a final concentration of 20% *w/v*, as well as protease inhibitor cocktail mix (Thermo Fisher Scientific, Waltham, MA), and the samples were incubated on ice for 3 h. Samples were centrifuged at 13,500× *g* at 4 °C for 15 min. Afterwards, the supernatants were removed, and the pellets were washed 3 times with cold acetone. After each wash step, samples were centrifuged at 13,500× *g* at 4 °C for 10 min. The formed pellet was dissolved by heating at 95 °C for 5 min in solution containing 2% *w/v* SDS and 5 mM DTT. Protein concentrations were measured using a BCA Protein Assay Kit (Thermo Fisher Scientific, Waltham, MA, USA) according to the manufacturer’s instructions and measured at 562 nm with a microplate reader (Epock; BioTek, BioTek Instruments, Inc., BioTek, Winooski, VT, USA). For the detection of cytokine production, Western blotting against target interleukins was carried out in a total protein amount of 50 μg.

### 2.11. Statistical Analysis

qPCR results are presented as the mean and standard deviation (SD) of experiments in triplicates. MTT results are presented as the mean and standard deviation of experiments in hexaplicates (for each concentration). Trypan blue staining results are presented as mean ± SD of three independent measurements. The relative expression of all target proteins was measured using ImageJ [34] and are presented as mean ± SD of three independent experiments. Student’s *t*-test was used for testing the differences between target groups with the statistical significance set at *p* = 0.05. Formal analysis for data significance and graphs were made using GraphPad Prism (Version 8.0.1).

## 3. Results

### 3.1. Transient Transfetion of A549 Cells with ORF3a and/or GFP mRNA

**Figure 1:**
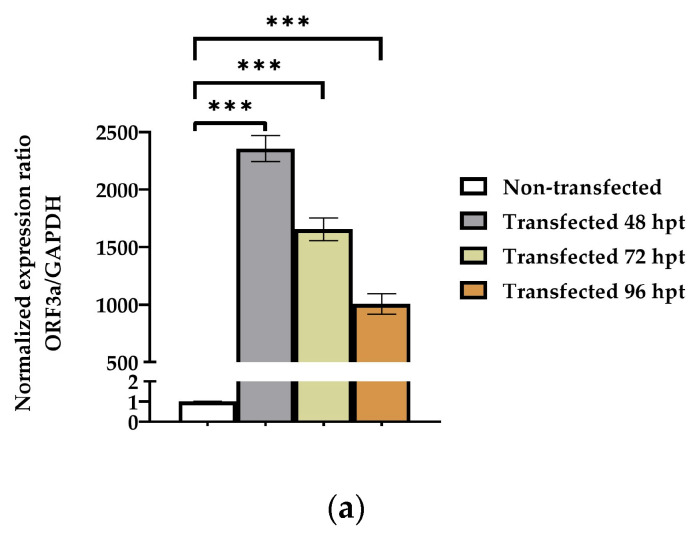

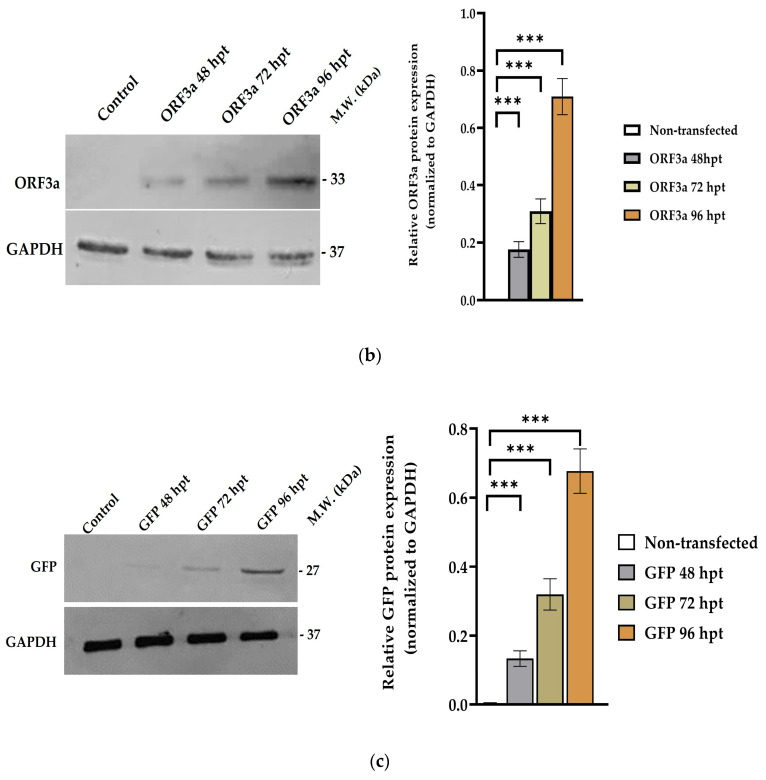
Transient transfection of A549 cells with ORF3a-mRNA. (**a**) Evaluation of *ORF3a* mRNA levels at 48, 72 and 96 h post-transfection by real-time PCR. *ORF3a* mRNA levels were normalized to GAPDH gene. (**b**) Detection of recombinant ORF3a protein levels 48, 72 and 96 h post-transfection by Western blotting. For the detection of recombinant ORF3a, a primary polyclonal anti-His tag antibody was used. ORF3a protein expression normalized to GAPDH protein levels. (**c**) Detection of Green Fluorescent Protein (GFP) protein levels 48, 72 and 96 h post-transfection by Western blotting. For the detection of GFP, a primary polyclonal anti-GFP antibody was used. GFP protein expression normalized to GAPDH protein levels. The notation (***) indicate statistically significant differences (*p* ≤ 0.001) compared to control cells.

Given that the respiratory system is the main tissue affected by COVID-19, *ORF3a* mRNA has been introduced into A549 lung carcinoma epithelial cells. Transfection of these cells was demonstrated by evaluating the relative expression of ORF3a at the mRNA and protein levels. As shown in Figure 1, the maximum ORF3a protein expression was observed 96 h post-transfection, and this time point was selected for the investigation of the ORF3a mechanisms of action, as described below. Similarly, transfection of A549 cells with *GFP’s* mRNA was carried out and used as a control. The protein levels of transfected GFP protein are shown in Figure 1c.

### 3.2. SARS-CoV-2 ORF3a Activates the PERK-eIF2a-ATF4 Branch of UPR

**Figure 2:**
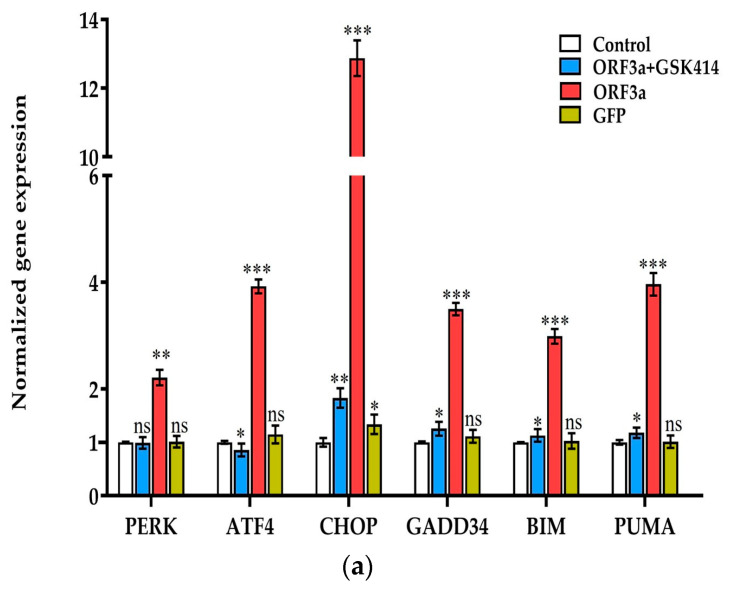

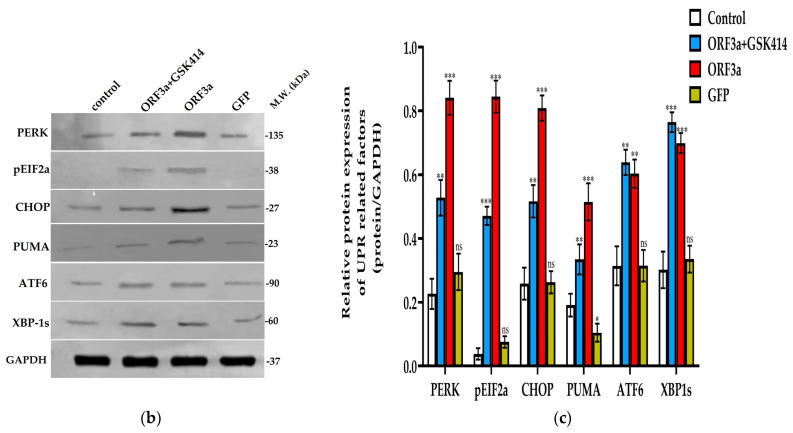
Evaluation of ORF3a-dependent UPR activation and its executioners at transcription and protein level. A549 cells were transfected with 0.5 μg of ORF3a (ORF3a group) or GFP (GFP group) mRNA (t = 0 h). After 48 h (t = 48 hpt), cells transfected with *ORF3a* mRNA were treated with 1000 nM of GSK2606414 for another 48 h (t_total_ = 96 hpt, ORF3a + GSK414 group). (**a**) Detection of PERK signaling pathway mediators and BH3-only genes; mRNA levels by qPCR in the control, GSK414, ORF3a and GFP groups. The expression levels of all target genes were evaluated at 96 hpt and were normalized to GAPDH levels. (**b**,**c**) Western-blot confirmation of selected UPR-involved and BH3-only proteins in the control, GSK414, ORF3a and GFP groups. GAPDH was used as housekeeping protein. The relative protein expression levels were calculated at 96 hpt for all groups using ImageJ by measuring band intensities in different (n = 3) blots and are represented in bar charts as mean ± SD. The relative protein expression of all target proteins is normalized to GAPDH levels Asterisks (*), (**), and (***) indicate statistically significant differences (*p* ≤ 0.05, *p* ≤ 0.01, and *p* ≤ 0.001, respectively) compared to control cells, while “ns” indicates non-significant differences.

The role of PERK branch in UPR upon cell transfection with ORF3a was investigated as follows: At first, the potential of ORF3a to activate UPR was examined by Western blotting against UPR-involved proteins such as PERK, XBP-1s (an IRE1 executioner) and ATF6 (Figure 2). ORF3a activates all three branches of UPR but our study focuses on PERK one due to its overexpression compared to ATF6 and IRE1a branches. Furthermore, we examined the role of PERK signaling pathway activation during ORF3a transfection. It is demonstrated that the mRNA levels of *PERK, ATF4*, and *CHOP* were notably upregulated (Figure 2a). The activation of the PERK pathway is proved via eIF2a phosphorylation in transfected cells (ORF3a group) compared to the control group (Figure 2b). Moreover, ATF4 upregulates the transcription of the pro-apoptotic transcription factor CHOP, an executioner of the PERK pathway, and therefore, that of the pro-apoptotic, BH3 only proteins, *BIM* and PUMA. CHOP and PUMA overexpression in mRNA (Figure 2a) as well as in protein levels (Figure 2b) was observed for both factors in A549 transfected cells compared to control. Additionally, *GADD34*, one of the few mRNAs that can be translated in the presence of phospho-eIF2a and functions in a feedback loop to either restore the general mechanism of protein translation and promote cell recovery from UPR or to enable the translation of apoptotic proteins, was found to be upregulated (Figure 2a). In GFP group little or non-significant differences of target genes and proteins expression, compared to the control group, are observed which clearly points out that UPR branches of ER stress aren’t “switched on” in GFP group (Figure 2) and therefore the observed activation is specific and is attributed to ORF3a transfection. To further investigate the implication of ORF3a in PERK pathway of UPR, cells were treated with GSK2606414, a specific inhibitor of PERK phosphorylation. The cytotoxic effects of 250 nM, 500 nM, 1000 and 1250 nM GSK2606414 in A549 cells were evaluated by MTT assay at 24 and 48 h after treatment (Figure S2). Considering cell viability, we demonstrated that 1000 nM GSK2606414 was the optimal concentration to retain sufficient cell viability and investigated whether PERK inhibition can restrain ORF3a-induced compilations discussed throughout the manuscript. A549 cells were transfected with *ORF3a* mRNA for 48 h and then treated with GSK2606414 (1000 nM) for another 48 h. The results of RT-qPCR showed that the mRNA levels of PERK axis executioners were significantly decreased in the ORF3a + GSK414 group (Figure 2a and Figure S3). Decreased phosphorylation of the eIF2a translation factor, as well as reduced protein expression of PERK, CHOP and PUMA in GSK2606414-treated cells (ORF3a + GSK414 group) were also observed (Figure 2b), indicating the inhibitory effect of GSK2606414. Expression of cleaved ATF6 and XBP-1s doesn’t show significant difference between ORF3a and ORF3a + GSK414 group (Figure S4).

### 3.3. SARS-CoV-2 ORF3a Promoted the Expression of Apoptotic Biomarkers

**Figure 3:**
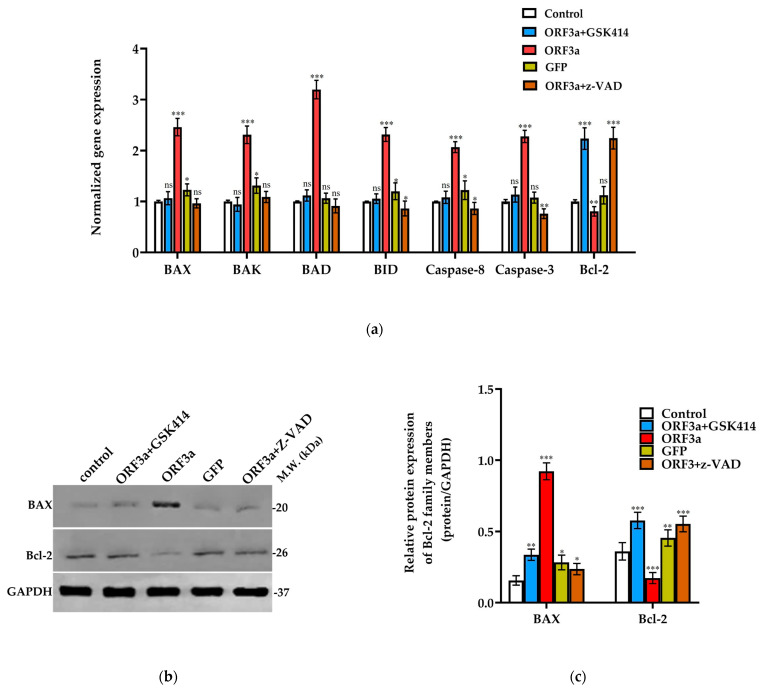

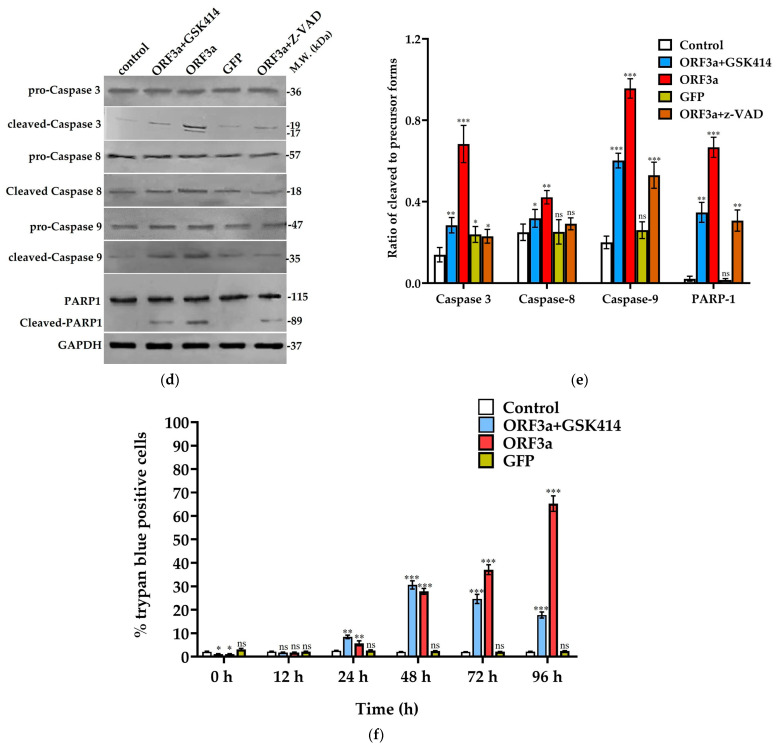

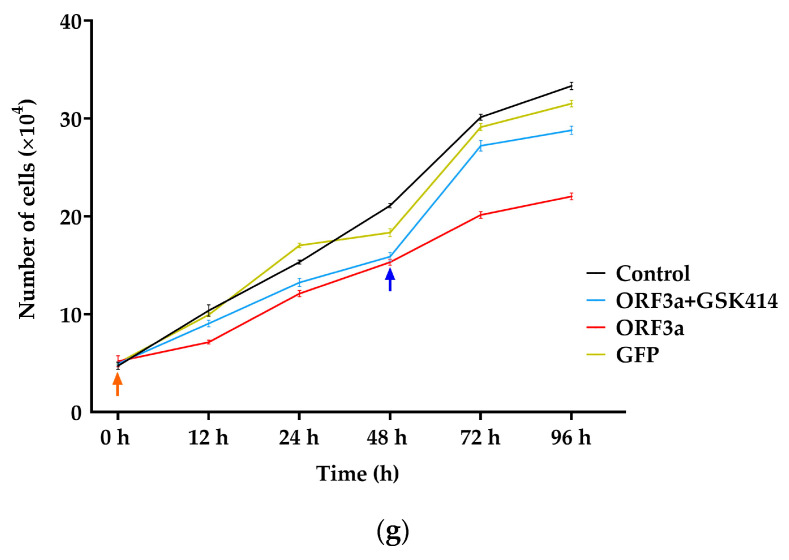
Investigation of ORF3a implication in apoptosis. A549 cells transfected with 0.5 μg of ORF3a (ORF3a group) or GFP (GFP group) mRNA (t = 0 h) were treated 48 h post-transfection (t = 48 hpt), either with 1000 nM of GSK2606414 (ORF3a + GSK414 group) or 50 μΜ z-VAD (ORF3a + z-VAD) for another 48 h (t_total_ = 96 hpt). (**a**) Relative quantification of the mRNA levels at 96 hpt of pro-apoptotic, anti-apoptotic and caspase family markers in the control, GSK414, ORF3a, GFP and z-VAD groups. The normalization of Ct values was performed against *GAPDH* gene. (**b**,**c**) Western blot confirmation of BAX and Bcl-2 protein expression in the control, GSK414, ORF3a, GFP and z-VAD groups at 96 hpt. (**d**,**e**) Western blotting against caspase-3, -8, -9 (precursor and cleaved forms) and PARP-1 (full length and cleaved form) in protein extracts of the control, GSK414, ORF3a, GFP and z-VAD groups. The bar charts next to each Western blot group photo represent the relative protein expression of the target proteins at 96 hpt after quantification of band intensities in the blots, using the ImageJ 1.53t software. The relative protein expression of all apoptotic biomarkers is normalized to GAPDH protein levels. (**f**,**g**) Evaluation of cell death kinetics and growth rate during the experimental setup for the control, GSK414, ORF3a, and GFP using Trypan Blue Dye Exclusion Assay. The time point of transfection (indicated with an orange arrow) was used as a starting time point (0 h). Measurements were carried out at specific time points 0, 12, 24, 48, 72 and 96 h post-transfection. In ORF3a + GSK414 group, A549 cells transfected with *ORF3a* mRNA were treated with GSK2606414 inhibitor 48 h post-transfection (indicated with a blue arrow). The data are presented as the mean ± SD values (n = 3). Asterisks (*), (**), and (***) indicate statistically significant differences (*p* ≤ 0.05, *p* ≤ 0.01, and *p* ≤ 0.001, respectively) compared to control cells while “ns” indicates non-significant differences.

Considering the upregulation of ER stress executioners of the PERK branch and that of pro-apoptotic BH3-only genes, we investigated whether ORF3a could induce apoptosis in A549 cells via the canonical PERK-eIF2a-ATF4 pathway. The role of ORF3a in inducing cell death was evaluated by quantifying the relative expression of apoptotic biomarkers in the presence or absence of *ORF3a* mRNA. Specifically, as shown in Figure 3a, we evaluated the expression of *BID*, *BAD*, *BAK*, *BAX*, *CASPASE8*, *CASPASE3* and *BCL-2*. Based on our findings, upregulation of apoptosis-related genes and downregulation of Bcl-2 was detected. We also observed an overexpression of BAX, a Bcl-2 pro-apoptotic family member, compared to Bcl-2 downregulation, in the ORF3a group (Figure 3b,c). Another proof of the apoptotic signaling of ORF3a was the detection of caspase-3, caspase-8, caspase-9 and PARP1 cleaved forms, which are primary executors of apoptotic death (Figure 3d,e). Cells transfected with GFP’s mRNA show similar expression profile as the control group. The apoptotic role of ORF3a in A549 transfected cells was also proved by measuring the cell death and growth rate with the Trypan Blue Dye Exclusion Assay (Figure 3f,g and Figure S8). As shown in Figure 2a and Figure 3a, a downregulation in the mRNA levels of BH3-only and Bcl-2 pro-apoptotic genes but a significant upregulation of Bcl-2 in the ORF3a + GSK414 group were demonstrated. The qPCR results are consistent with the protein expression profile of BAX and Bcl-2 in the respective groups. Thus, it is indicated that transfected cells treated with the PERK inhibitor (ORF3a + GSK414 group) are less susceptible to apoptosis than the untreated group (ORF3a group) (Figures S5 and S6). Interestingly, the ORF3a + GSK414 group showed a marked decrease in cleaved-forms of Caspase-3, -8, -9 and PARP-1 (Figure 3b and Figure S7). These data reveal that PERK inhibition reduces apoptosis induced by the viral protein ORF3a in transfected lung epithelial, A549, cells. For further validation of apoptosis inhibition, transfected cells were treated with z-VAD (ORF3a + z-VAD). Indeed, as shown in Figure 3a–e, treatment of transfected cells with 50 μΜ z-VAD favors cell survival by reducing the levels of pro-apoptotic BAX protein and inhibiting the cleavage and therefore activation of studied caspases and PARP-1 (Figure 3a–e). Finally, cell survival was figured by the elevated expression of Bcl-2 in both mRNA and protein level (Figure 3a–c).

### 3.4. SARS-CoV-2 ORF3a Subcellular Localization in A549 Is Consistent with PERK Dependent Apoptosis Mechanism of Action

**Figure 4:**
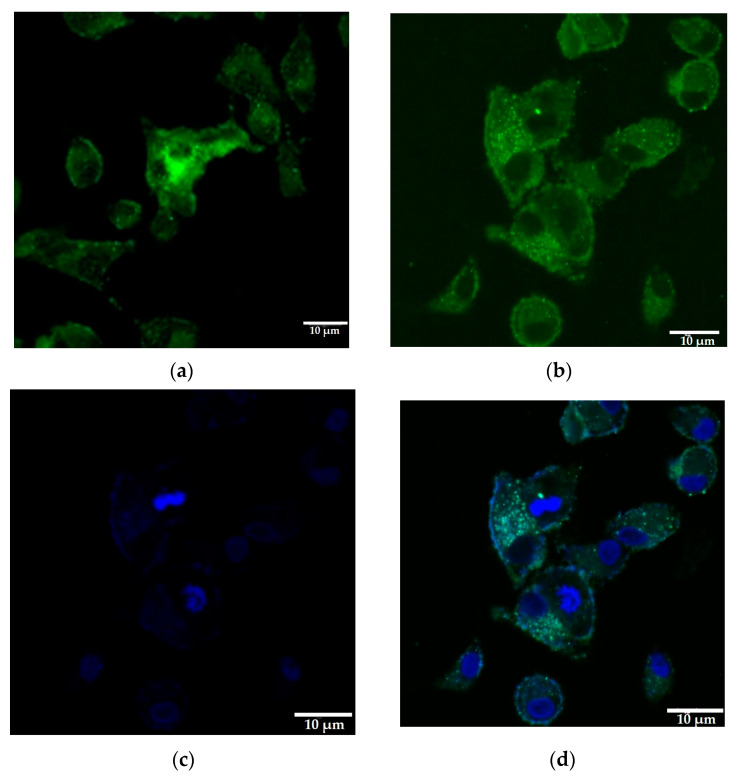
Subcellular localization of ORF3a-GFP fusion protein in A549 cells. A549 cells transiently transfected with the recombinant plasmid were fixed on borosilicate glass coverslips with 4% paraformaldehyde and relative fluorescence of samples were studied at 48 hpt. (**a**) A549 cells transfected with pEGFP-N1 (control), (**b**) A549 cells transfected with pEGFP-N1-ORF3a, (**c**) DAPI staining of A549 nucleus area, (**d**) merge of pEGFP-N1-ORF3a (**b**) and DAPI (**c**) images. All images were taken with a confocal microscope at 40× magnification and their scale was set to 10 μm.

The subcellular localization of SARS-CoV-2 ORF3a protein was examined by transient transfection of A549 cells with the pEGFP-N1-ORF3a recombinant plasmid. Cells were fixed with paraformaldehyde 48 h post-transfection and observed using confocal microscopy. As shown in Figure 4, ORF3a-GFP was mainly localized in the cytoplasm, leaving the nucleus vacant. It can be presumed that ORF3a is mainly localized in the membranous structures of the cell, such as the endoplasmic reticulum, lysosomes and mitochondria.

### 3.5. ORF3a Triggers Inflammatory Response of Lung Epithelial Cells

**Figure 5:**
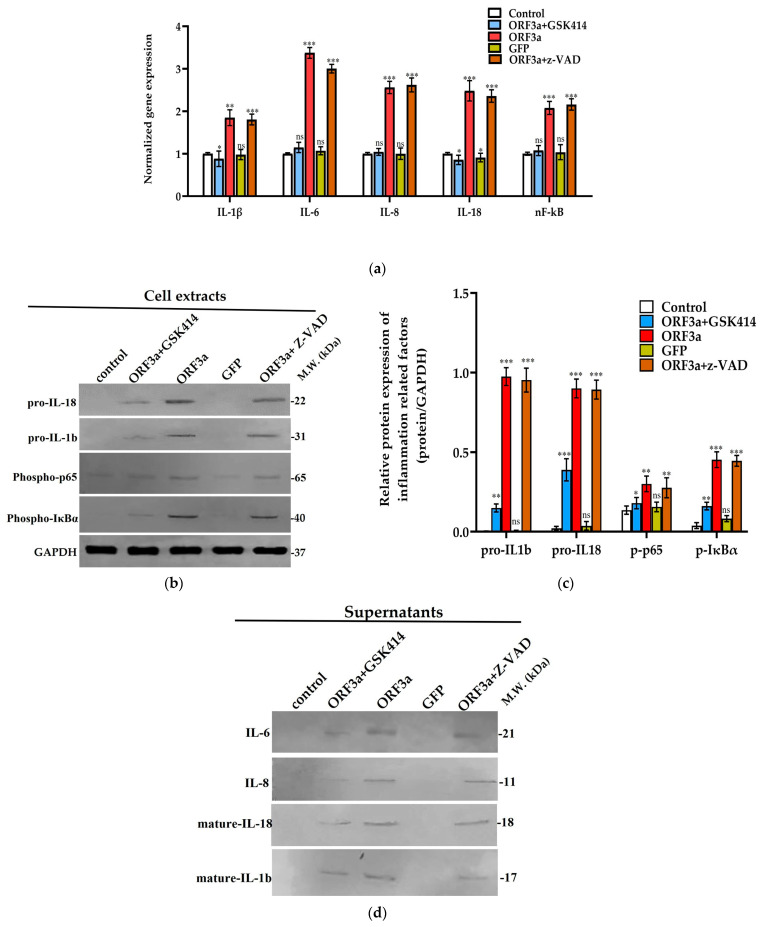
Investigation of ORF3a role in inflammatory response of lung epithelial cells. A549 cells were transfected with 0.5 μg of ORF3a (ORF3a group) or GFP (GFP group) mRNA (t = 0 h). After 48 h (t = 48 hpt), cells transfected with *ORF3a* mRNA were treated either with 1000 nM of GSK2606414 or 50 μΜ z-VAD for another 48 h (t_total_ = 96 hpt, ORF3a + GSK414 group). (**a**) Assessment of mRNA expression of pro-inflammatory cytokine genes (*IL-1b*, *IL-6*, *IL-8*, *IL-18*) as well as *NFκB* in the control, GSK414, ORF3a, GFP and z-VAD groups at 96 hpt. GAPDH was used as a housekeeping gene. (**b**,**c**) Western blot confirmation of IL-1b and IL-18 precursor forms as well as phospho IκB and phospho p65 in cell extracts of the control, GSK414, ORF3a, GFP and z-VAD groups at 96 hpt. Next to each photograph are included bar blots indicating the relative protein expression of all target proteins after quantification of band intensities. The data are presented as the mean ± SD values (n = 3). Asterisks (*), (**), and (***) indicate statistically significant differences (*p* ≤ 0.05, *p* ≤ 0.01, and *p* ≤ 0.001, respectively) compared to control cells. The relative protein expression of all inflammation markers is normalized to GAPDH protein levels. (**d**) Western blotting at 96 hpt against IL-1b and IL-18 mature forms as well as IL-6 and IL-8 in cell culture supernatants of the control, GSK414, ORF3a, GFP and z-VAD groups after TCA/acetone precipitation while “ns” indicates non-significant differences.

An interesting finding of our study was that the viral protein ORF3a can directly or indirectly induce the expression of pro-inflammatory cytokines. We examined the expression of these cytokines by evaluating the mRNA levels of *IL-1*, *IL-6*, *IL-8*, *IL-18* and *NFκB* in lung epithelial cells and found upregulation of all inflammatory factors mentioned above (Figure 5a). Moreover, we examined the protein levels of precursor forms of IL-1 and IL-18 (Figure 5b,c), markers of systemic inflammation, as well as the secreted mature forms after TCA precipitation of cell culture supernatants. We also found an increased expression of IL-6 and IL-8 in cell culture supernatant of ORF3a group cells (Figure 5d). The expression of these cytokines is consistent with the activation of NFκB, as indicated by the detection of the phosphorylated forms p65, an NFκB subunit, and IκΒα, inhibitor of NFκB (Figure 5b,c). GFP group shows non-significant changes in their mRNA and protein expression profile (Figure 5). Treatment of transfected A549 cells with GSK2606414 ameliorates the ORF3a-induced inflammatory response by reducing the expression of intracellular and extracellular pro-inflammatory factors, mentioned in Figure 5. Furthermore, the ORF3a + GSK414 group shows decreased phosphorylation of p65 subunit of ΝFκΒ and ΙκΒα, indicating a less active NFκΒ (Figures S9 and S10). In contrary to the ORF3a + GSK414 group, treatment of transfected cells with z-VAD does not affect the levels of phospho p65 and IκΒα as well as these of the intracellular pro-inflammatory cytokines. Concerning the secreted cytokines, the ORF3a + z-VAD group displays just a slight decrease only in mature IL-1b and 18 levels, without affecting ex-pression of IL-6 and IL-8.

## 4. Discussion

The accessory protein ORF3a of SARS-CoV-2 is a multifunctional protein linked to virulence, infectivity, and virus–host cell interactions [35]. Most of our knowledge about SARS-CoV-2 proteins is derived from studies of the homologous proteins of SARS-CoV and MERS-CoV. Studies on the 3a protein of SARS-CoV, which shows 72% homology to the SARS-CoV-2 ORF3a protein, have demonstrated that the 3a protein of SARS-CoV can cause ER stress and suppress the type 1 interferon response via PERK activation. SARS-CoV ORF3a, ORF6, ORF8, and spike (S) proteins have been reported to induce ER stress [36]. Minakshi et al. (2009), for the SARS-CoV homolog protein, suggested that ORF3a activates p38 MAPK and is pro-apoptotic by inducing ER stress [37]. In addition, early studies on the SARS-CoV-2 ORF3a protein demonstrated the role of ORF3a in apoptosis in general, without specifying the exact mechanism by which it is involved [5]. Additionally, recent findings have shown that SARS-CoV-2 ORF3a can promote the activation of all UPR branches, supporting the notion that ORF3a-induced autophagy is dependent on the ATF6 and IRE1 signaling pathways but not the PERK pathway. However, the complex interaction between the UPR and autophagy during ORF3a transfection requires further investigation [36]. Thus, the exact role of PERK branch of UPR in SARS-CoV-2 infections remains unclear.

Based on the latter statement and on the fact that ORF3a is a multifunctional protein, we decided to unravel the role of ORF3a-dependent PERK activation. In this study, the experimentally supported mechanism by which ORF3a induces pyroptosis and apoptosis in lung epithelial cells was discussed. First of all, it is important to acknowledge that our approach involved replicating virus infection as closely as possible. For this purpose, we utilized an in vitro synthesized transcript of ORF3a, which was incorporated into a commercially available lipid nano carrier for efficient cell transfection. The comprehensive demonstration of this process can be found throughout the manuscript. By simulating the mechanism of infection, we believe to have limited artifacts related with virus cell entrance. To eliminate any potential confounding effects on the studied signaling pathways that could arise from mRNA transfection, we implemented Green Fluorescent Protein mRNA as a control. This measure was taken to ensure that any positive results observed were specifically attributed to the signaling pathways under investigation.

The first thing to prove was UPR activation. Indeed, ORF3a causes the increased expression of UPR-involved proteins (PERK, XBP-1s (IRE1 executioner) and cleaved form of ATF6), with notable the overexpression of PERK kinase protein. The activation of PERK is further demonstrated by detecting the phosphorylation levels of eIF2a and the upregulation, in mRNA level, of PERK-eIF2a-ATF4 axis executioners and that of BH3-only protein family (*BIM, BAD, PUMA* etc.) in ORF3a transfected A549 cells. BH3-only proteins are encoded and activated during ER stress when elevated expression of *CHOP* is observed [38,39]. All qPCR experiments are constituent to Western blotting against some of the target proteins, such as these involved in UPR (PERK, CHOP, PUMA, XBP-1s and cleaved ATF6) and apoptosis (PUMA). Furthermore, the mechanism by which ORF3a induces apoptosis was investigated.

Particularly, as mentioned previously, the relative expression of specific apoptotic markers at the mRNA level has been evaluated and the apoptotic character of ORF3a was verified by detecting cleavage of caspase family members (caspase-3, -8, and -9) and PARP-1 as well as BAX and Bcl-2 expression. Thus, the observed proteolytic cleavage of caspases (-3, -8, and -9), PARP-1, overexpression of BAX (a representative pro-apoptotic Bcl-2 family member protein) and decreased expression of Bcl-2, an anti-apoptotic protein, in transfected cells compared to the control group, are solid proof of the apoptotic character of the viral protein ORF3a.

In addition, based on findings regarding the apoptotic character of other proteins or therapeutic compounds, such as chemotherapeutics, the BAX/Bcl-2 expression ratio at the mRNA level was studied [40,41,42]. Specifically, a high value of this ratio, namely a greater expression of Bax, indicates a breakdown of the mitochondrial membrane potential, leading to the release of cytochrome c, and consequently, apoptosis in a mitochondrial-dependent manner. Indeed, in the transfected group (ORF3a group), a ratio of 3.05 was calculated, indicating that A549 cells were susceptible to cell death. The molecular phenomena described above are consistent with the protein expression profile of ORF3a group compared to the control (BAX overexpression, reduced Bcl-2 expression, caspases and PARP-1 cleavage as well as PUMA elevated expression). Considering that ORF3a is localized in the cytoplasm and specifically in the membranous structures of the cell, we strongly suggest that ORF3a mainly induces apoptosis via the mitochondrial pathway. The upregulation of BID and caspase-8 as well as the proteolysis of the latter indicate the activation of the extrinsic pathway of apoptosis. However, the exact mechanism by which ORF3a induces the extrinsic pathway remains unclear and more experiments are needed for a more descriptive mechanism to be elucidated.

One of the main characteristics of severe COVID-19 cases is the hyperinflammation syndrome or cytokine storm, which can provoke tissue damage, acute respiratory distress syndrome (ARDS) and multi-organ failure [43]. As the first line of mechanical and chemical defense against different types of intruders, epithelial cells can produce trace amounts of cytokines [44]. In our project, it is shown that the inflammatory response of lung epithelial cells is dependent to the upregulation of *NFκB* as well as the activation of that pro-inflammatory factor in protein level by detecting the phosphorylation of p65 subunit of and that of IκΒα. These two events are interpreted as a transcriptionally active NFκB factor that can promote expression of pro-inflammatory factors, and secondly, the phosphorylation of IκΒα acts as an initial signal for degradation of that NFκB inhibitory factor. The activation of NFκB is seemed to be accompanied to an upregulation of inflammatory cytokines mRNA and protein levels of the respective interleukins. The presence of pro-IL-1b and pro-IL-18 in cell extracts, as well as the detection of mature IL-1b and 18, IL-6 and IL-8 in cell culture supernatants, are solid evidence of an ORF3a-induced inflammatory response.

**Figure 6:**
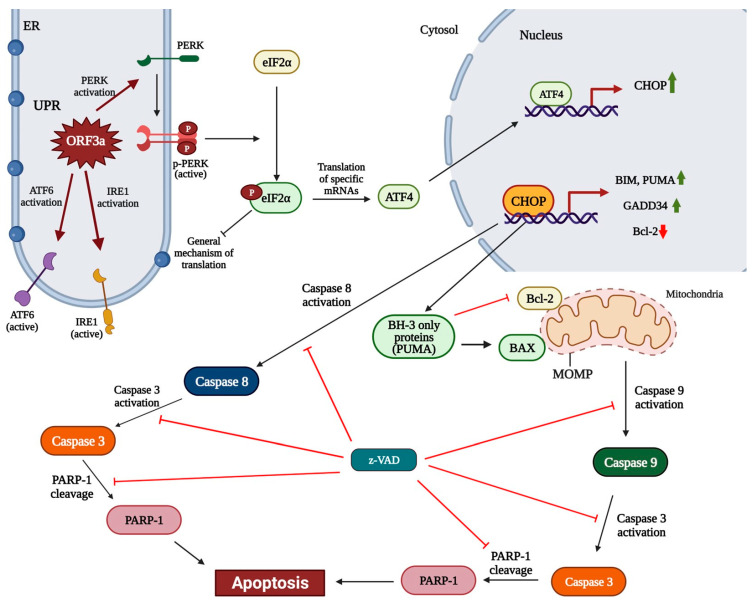
ORF3a functions as an “endoplasmic reticulum stressor” causing UPR (Unfolded Protein Response) and manipulating PERK to initiate apoptosis. Indeed, ORF3a activates all three branches of UPR, ATF6, IRE1 and PERK, leading cells to a pathological state. In particular, ORF3a seems to recruit PERK branch in order to exhibit its apoptotic character. Activated PERK phosphorylates translation initiation factor, eIF2a, leading to shut down of the general mechanism of translation and allowing the translation of specific mRNAs such as this of ATF4 [53,54,55]. ATF4 induces the transcription and translation of the pro-apoptotic transcription factor CHOP (C/EBP homologous protein). Then, CHOP orchestrates apoptosis through Bcl-2 down-regulation, caspase family members recruit and elevation of BH3-only (pro-apoptotic) proteins expression. CHOP can activate caspase 8, which in turn induces caspase 3 cleavage, a mid-apoptosis stage event. Furthermore, CHOP induces the expression of BH3-only pro-apoptotic proteins such as PUMA that induces the expression and activation of BAX and inhibits Bcl-2 from displaying its anti-apoptotic character. BAX is a pro-apoptotic protein that can provoke mitochondrial outer membrane permeabilization (MOMP), when Bcl-2 is down-regulated or inhibited, leading to caspase 9 activation and thus caspase 3 cleavage. PARP-1 is a substrate of proteolytic cleavage by activated caspase 3. PARP-1 cleavage is a hallmark of later stages of apoptosis as the cleaved enzyme is inactive and cannot repair damage in DNA strands as cells are severely and irreversibly damaged. Z-VAD is a pan-caspase inhibitor, which binds to caspase catalytic site, retains them to their inactive form and blocks apoptosis. Treating ORF3a transfected cells with z-VAD can inhibit caspases-3, -8 and -9 activation and counteract the apoptotic fate of the cells. The green arrows indicate upregulated genes, while red arrows indicate down-regulation of target genes. Created with BioRender.com (accessed on 16 May 2023).

The interconnection between ORF3a-induced PERK activation and previously described compilations was demonstrated by treating transfected cells with GSK2606414, a PERK-specific inhibitor. Indeed, treatment of transfected cells 48 h after transfection resulted in a significant decrease in the transcriptional and protein level of PERK axis markers, such as *ATF4* (mRNA level), CHOP and PUMA (mRNA and protein level). Moreover, the reduced phosphorylation levels of eIF2a indicated successful inhibition of the PERK axis in the ORF3a + GSK414 group cells. The other two branches of UPR are unaffected after treatment with PERK inhibitor, based on Western blotting against executioners of these pathways, indicating that all observed complications are due to PERK activation. Taken together, treatment of *ORF3a* mRNA transfected A549 cells with GSK2606414 can terminate ER stress caused by PERK activation. Similarly, it is observed that mRNA levels of inflammatory cytokines and apoptotic markers were significantly downregulated in the ORF3a + GSK414 group compared to ORF3a group. The suppression of apoptosis is also supported by the calculated BAX/Bcl-2 ratio of 0.48 and the constituent changes in protein levels of these two proteins (Figure 3b,c). An intriguing observation in Figure 3 is that z-VAD (ORF3a + z-VAD group) blocked mRNA levels of apoptotic proteins and BAX protein levels increase, but pro-caspase levels remained high. These expression differences could be a secondary effect due to z-VAD promoting cell survival. This is also consistent with the increase in levels of the anti-apoptotic factor Bcl-2 shown in our results (Figure 3a,b). Based on the literature data [45,46,47,48,49,50], Bcl-2 seems to influence the function and consequently the expression of pro-apoptotic factors. Thus, the changes in mRNA and/or protein expression levels may not be a direct effect of z-VAD but may be due to the restoration of Bcl-2 to normal levels. Lastly, z-VAD appears to inhibit caspase-dependent apoptosis, but in the presence of an appropriate (cytotoxic) stimulus induces other forms of cell death such as necroptosis through activation of the RIPK1-RIPK3 complex [51,52]. This is associated with an inability to form the RIPK1-activated caspase8 complex, which induces extrinsic apoptosis, in the presence of z-VAD. This, combined with the low expression levels of apoptotic proteins, may suggest that ORF3a could induce (to a lesser extent) other forms of cell death beyond apoptosis, which needs further investigation. The proposed mechanism of ORF3a-“induced” apoptosis is shown in Figure 6.

Lastly, it has been shown that ORF3a can induce inflammation in lung cells in a PERK-dependent manner. Previous works demonstrated that ORF3a viroporin can activate NLRP3 inflammasome in lung epithelial cells causing the secretion of mature IL-1b and IL-18 [19]. Moreover, there are findings supporting that the general mechanism of translation inhibition due to PERK activation prevents the binding of IkB to NFκB, allowing the latter to translocate to the nucleus and trigger the expression of inflammatory cytokines [56,57]. Based on our data, we propose that in a SARS-CoV-2 infection there is a crosstalk among ER stress and inflammatory response in lung epithelial cells through ORF3a-induced PERK activation. In particular, inhibition of PERK by treating cells with GSK2606414 can reduce the phosphorylation levels of p65 subunit of NFκB and IκΒα, an inhibitory factor of NFκB. Furthermore, treatment with GSK2606414 can ameliorate all effects mentioned above. Solid proof that GSK2606414 can counteract ORF3a-induced inflammation and pyroptosis is the downregulation of all inflammatory cytokines, as well as that of NFκB, and the reduced protein expression of IL-1b and IL-18 precursors in cell extracts. The ORF3a + GSK414 group also shows reduced phosphorylation levels of p65 subunit as well as that of ΙκΒα, and results that show that inhibitor protein ΙκΒα can bind to NFκB and prevent p65 phosphorylation and therefore its activation. Consistent results are taken for cell culture supernatants, in which the protein levels of all studied pro-inflammatory interleukins are reduced. Further solid proof that ORF3a-provoked PERK activation is the direct cause of apoptosis, and the inflammatory response of A549 transfected cells are coming from treatment of transfected cells with z-VAD. Our results suggest that, although treatment with z-VAD (a pan-caspase inhibitor) is used for ameliorating the events mentioned above, the inhibitor can only restrain, to some extent, apoptosis but not inflammatory response. The only difference observed is that the z-VAD group shows a slight decrease in the expression of mature IL-1b and IL-18, but not those of IL-6 and -8 in cell culture supernatants; a fact that is consistent with the activation of these proteins by their proteolytic cleavage by caspase-1 [58,59,60].

**Figure 7:**
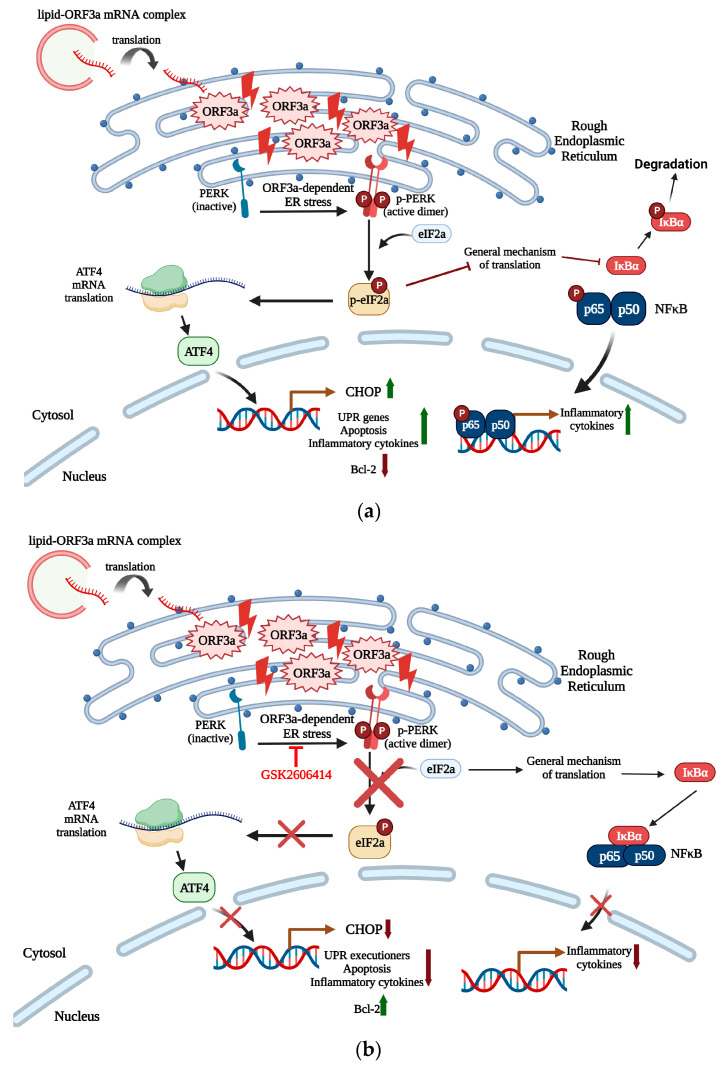
(**a**) Transfection of lung epithelial cells with ORF3a-mRNA induce ER stress and activates PERK. PERK-dependent UPR promotes phosphorylation of the translation factor eIF2a, inhibiting the general mechanism of translation and allowing the translation of specific mRNAs, such as ATF4. ATF4 then promotes the transcription of CHOP, a pro-apoptotic transcription factor that regulates the expression of GADD34, BH3-only proteins, and mitochondria-related apoptotic proteins. CHOP also inhibits the action of anti-apoptotic proteins, such as Bcl-2. GADD34 acts in a feedback loop to dephosphorylate eIF2a and to promote translation initiation. This effect induced GADD34-induced translation of apoptotic proteins. The effect of ORF3a-induced PERK activation involves the expression of inflammatory cytokines in an NFκB manner. Inhibition of translation prevents the binding of inhibitor IκB to NFκB, leading to p65 subunit phosphorylation and translocation of the latter to the nucleus and promoting the expression of inflammatory cytokines such as interleukins-1b, -6, -8, and-18. Moreover, IκΒα phosphorylation acts as an initiator signal for subsequent degradation of ΙκΒα. (**b**) Treatment of ORF3a-transfected cells with the PERK-selective inhibitor GSK2606414 prevented PERK activation and eIF2a phosphorylation, leading to cell survival signaling and an elevated expression of Bcl-2. In addition, PERK axis executioners, such as CHOP and PUMA, are downregulated and “under-expressed”, preventing ER stress and therefore apoptosis. Inhibition of PERK prevents NFκB activation and thus nuclear translocation and the expression of inflammatory cytokines and therefore cytokine storm. The green arrows indicate upregulated genes, while red arrows indicate down-regulation of target genes. Created with BioRender.com (accessed on 11 April 2023).

Taken together, our results suggest that ORF3a promotes inflammatory response and apoptosis through PERK activation. In particular, inflammatory response is dependent to NFκB activation through PERK activation and the subsequent inhibition of general translation mechanism. Thus, IκΒα, an NFκB inhibitor, is susceptible to phosphorylation, a post-translational modification that provokes its degradation, allowing p65 subunit of NFκB to be phosphorylated and become transcriptionally active [61,62,63]. On the other hand, apoptosis is triggered through PERK, CHOP and other pro-apoptotic proteins (BAX and PUMA) overexpression as well as caspases and PARP-1 cleavage. The reduced expression of Bcl-2 in ORF3a group is another proof that transfected cells are susceptible to cell death. Both inflammation and apoptosis observed in ORF3a transfected lung epithelial cells can be ameliorated by treating cells with a PERK specific inhibitor, GSK2606414. The proposed ORF3a mechanism of action and its inhibition are shown in Figure 7.

## 5. Conclusions

Although it has been over 3 years since the pandemic outbreak of SARS-CoV-2, there has not been much work on developing a novel therapeutic approach for both COVID-19 and long COVID symptoms. Importantly, unraveling the virus–host interaction is essential to develop effective antiviral therapies, such as drugs or drug cocktails targeting one or more proteins of SARS-CoV-2. This project demonstrated that the viral protein ORF3a could be a crucial cause of lung damage and/or hyperinflammation of the respiratory tissue during SARS-CoV-2 infection. In addition, we elucidated that PERK inhibitors can be potentially used as small molecular drugs to target the effects caused by the novel coronavirus 3a protein. Taken all in consideration, an intriguing question emerges: Can PERK-induced UPR be a potential target for the development of novel “anti-COVID19” drugs?

## Data Availability

The data presented in this study are available on request from the corresponding author.

## Supplementary Materials

The following supporting information can be downloaded at: https://www.mdpi.com/article/10.3390/biomedicines11061585/s1, Figure S1: Cloning of the ORF3a gene into the pT7-IRES-His-C DNA plasmid vector and *ORF3a* mRNA synthesis by in vitro transcription; Figure S2: Cytotoxicity assay (MTT) for A549 cells treated with PERK inhibitor, GSK2606414; Table S1: Primers for amplifying the ORF3a gene from pGBW-m4134093 and cloning it into the pT7-IRES-His-C DNA and pRGFP-N1 vector, respectively; Table S2: Oligonucleotide sequences for target genes amplification used in qPCR experiments. Primers indicated with (*) were designed using NCBI-Primer design tool (Primer BLAST); Figures S3–S10: Figures indicating statistically significant differences between ORF3a and ORF3a + GSK414 group. Next to each figure is indicated the corresponding one in the manuscript, in which the control and ORF3a groups are compared.
