# Supplementary material for: Inhibition of PERK Kinase, an Orchestrator of the Unfolded Protein Response (UPR), Significantly Reduces Apoptosis and Inflammation of Lung Epithelial Cells Triggered by SARS-CoV-2 ORF3a Protein
Source: Biomedicines. 2023 May 30;11(6):1585. doi: 10.3390/biomedicines11061585 (PMC10296123; doi:10.3390/biomedicines11061585)
Supplement: Supplementary file 1 [file biomedicines-11-01585-s001.zip › biomedicines-2396086-supplementary.pdf]

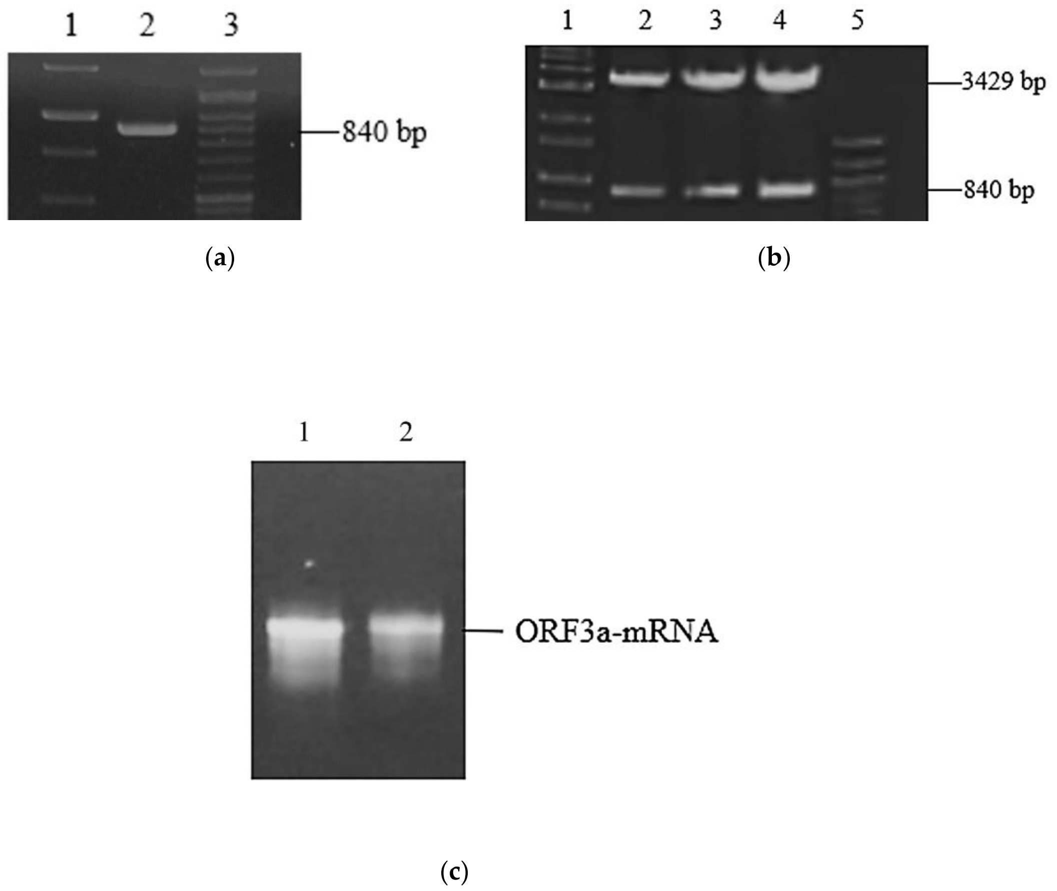

Figure S1: Cloning of the ORF3a gene into the pT7-IRES-His-C DNA plasmid vector and ORF3a mRNA synthesis by in vitro transcription. (a) PCR amplification of ORF3a (840 bp) using pGBW- m4134093 as DNA template (Lane 1: 1kb DNA ladder, Lane 3: 50 bp DNA ladder) (b) Recombinant pT7-IRES-His-C DNA-ORF3a double digested with NdeI and XbaI. The band of 3429 bp indicates the linearized pT7-IRES-His-C DNA, while ORF3a is demonstrated by the 840 bp band (Lane 1: 1kb DNA ladder, Lane 5: 100 bp DNA ladder). (c) Agarose gel elextrophoresis of ORF3a-mRNA bands before and after in vitro addition of poly(A) tail (Lane 1: mRNA (-)poly(A) tail, Lane 2: mRNA (+)poly(A) tail).

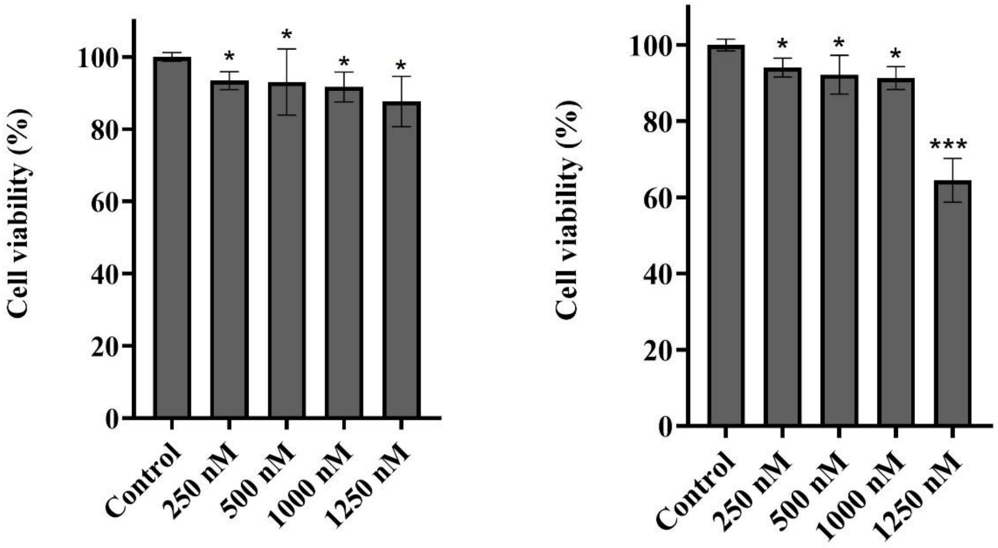

(**a**) (**b**)

Figure S2: Cytotoxicity assay (MTT) for A549 cells treated with PERK inhibitor, GSK2606414. Cell viability (a) 24 hours and (b) 48 hours after cells treatment with GSK2606414. For both time points, cells were treated with 0 (control), 250, 500,1000 and 1250 nM of inhibitor, respectively (* = $p < 0.05$, ** = $p < 0.01$, ***= $p < 0.001$).

Table S1: Primers for amplifying the ORF3a gene from pGBW-m4134093 and cloning it into the pT7-IRES-His-C DNA and pRGFP-N1 vector, respectively.

Target Gene	Forward primer (5'→3')	Reverse primer (5'→3')
ORF3a-pT7-IRES-His-C DNA	CCCCATATGGATTTGTTTATGAGAATC	CGCTCTAGAGGTAGAATCTAAGCC
ORF3a-pEGFP-N1	CCGCTCGAGATGGATTTGTTTATGAG	CGCGGATCCTTGGTAGAATCTAAGCC

Table S2: Oligonucleotide sequences for target genes amplification used in qPCR experiments. Primers indicated with (*) were designed using NCBI-Primer design tool (Primer BLAST).

Target Gene	Forward primer (5'→3')	Reverse primer (5'→3')	Ref.
ORF3a	GCAACGATACCGATACAAGCC	CCAGCAGCAACGAGCAAAA	[25]
GAPDH	GCACCGTCAAGGCTGAGAAC	TGGTGAAGACGCCAGTGGA	*
CASP8	AGAGTCTGTGCCCAAATCAAC	GCTGCTTCTCTCTTTGCTGAA	[26]
BID	CCTTGCTCCGTGATGTCTTTC	GTAGGTGCGTAGGTTCTGGT	[26]
BAK	ATGGTCACCTTACCTCTGCAA	TCATAGCGTCGGTTGATGTCG	[26]
BAD	CCCAGAGTTTGAGCCGAGTG	CCCATCCCTTCGTCGTCCT	[26]
BAX	GGGCCCACCAGCTCTGA	CCTGCTCGATCCTGGATGA	[27]
CASP3	CTAGCGGATGGGTGCTATTGT	CACGGATACACAGCCACAGG	*

BIM	ACCAAACCAAAGCCGTCATCA	GGAGCCAGTAAACGTATTGGAAG	[28]
BCL-2	CTTGACAGAGGATCATGCTGTAC	GGATGCTTTATTTCATGAGGC	[27]
CHOP	TGGAAGCCTGGTATGAGGAC	TGTGACCTCTGCTGGTTCTG	[29]
ATF4	CCCTTCACCTTCTTACAACCTC"	TTCACTGCCCAGCTCTAAAC	[30]
NF-KB	ATGGCTTCTATGAGGCTGAG	GTTGTTGTTGGTCTGGATGC	[31]
IL6	GCACTGGCAGAAAACAACCT	TCAAACTCCAAAAGACCAGTGA	[32]
IL1B	ATCACTGAACTGCACGCTCC	TTGTTCTCCATATCCTGTCCC	[31]
IL8	CGATGTCAGTGGATAAAGACA	TGAATTCTCAGCCCTCTTCAAAAA	[31]
IL18	GCCTAGAGGTATGGCTGTAA	GCGTCACTACACTCAGCTAA	[33]
PUMA	GACCTCAACGCACAGTACGAG	AGGAGTCCCATGATGAGATTGT	*
GADD34	CTGGCTGGTGGAAGCAGTAA	TATGGGGGATTGCCAGAGGA	*
PERK	ATAGGACAGAGGGGACAGAGTT	CGCAGTCTCAATCTCTGGACA	*

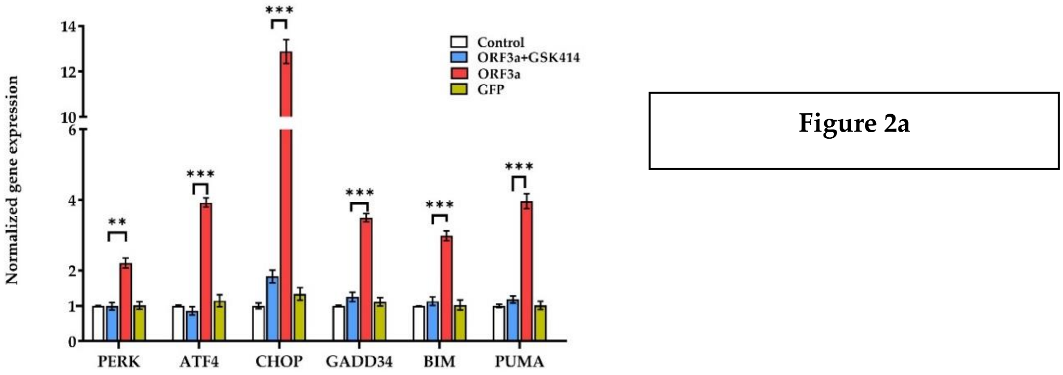

Figure S3: Detection of PERK signaling pathway mediators and BH3-only genes, mRNA levels by qPCR in control, ORF3a+GSK414, ORF3a and GFP group. The expression levels of all target genes were evaluated at 96 hpt and were normalized to GAPDH levels. Asterisks (*), (**), and (***) indicate statistically significant differences ($p \leqslant 0.05$, $p \leqslant 0.01$, and $p \leqslant 0.001$, respectively) between ORF3a and ORF3a+GSK414. Next to this figure is indicated the corresponding one in the manuscript, in which control and ORF3a group are compared.

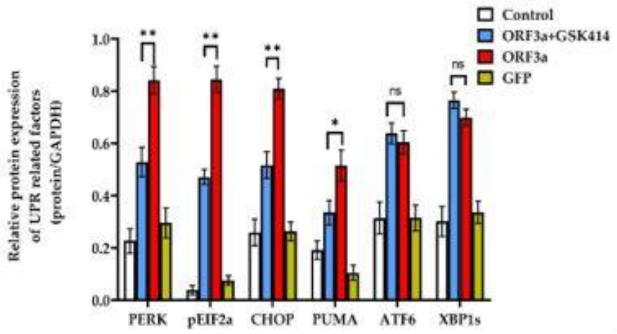

Figure 2c

Figure S4: The relative protein expression levels of selected UPR-involved and BH3-only proteins in control, ORF3a+GSK414, ORF3a and GFP group. The relative protein expression levels were calculated at 96 hpt for all groups using ImageJ by measuring band intensities in different (n=3) blots and are represented in bar charts as mean ± SD . The relative protein expression of all target proteins is normalized to GAPDH levels. Asterisks (*), (**), and (***) indicate statistically significant differences ($p \leqslant 0.05$, $p \leqslant 0.01$, and $p \leqslant 0.001$, respectively) between ORF3a and ORF3a+GSK414. Next to this figure is indicated the corresponding one in the manuscript, in which control and ORF3a group are compared..

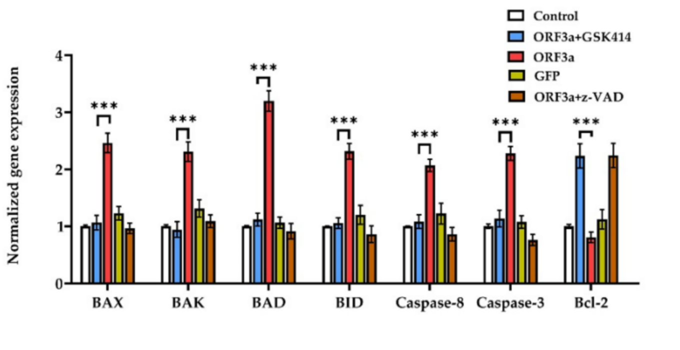

Figure 3a

Figure S5: Relative quantification of the mRNA levels at 96 hpt of pro-apoptotic, anti-apoptotic and caspase family markers in control, ORF3a+GSK414, ORF3a, GFP and ORF3a+z-VAD group. The normalization of Ct values was performed against GAPDH gene. Asterisks (*), (**), and (***) indicate statistically significant differences ($p \leqslant 0.05$, $p \leqslant 0.01$, and $p \leqslant 0.001$, respectively) between ORF3a and ORF3a+GSK414. Next to this figure is indicated the corresponding one in the manuscript, in which control and ORF3a group are compared.

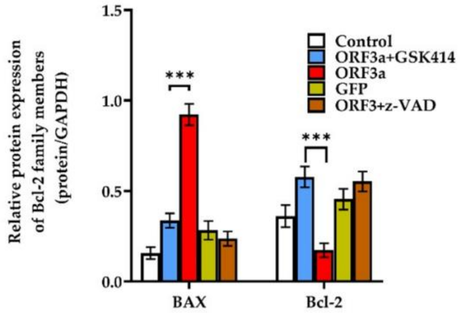

Figure 3c

Figure S6: The relative protein expression levels of BAX and Bcl-2 proteins in control, ORF3a+GSK414, ORF3a GFP and ORF3a+zVAD group. The relative protein expression levels were calculated at 96 hpt for all groups. The relative protein expression of all target proteins is normalized to GAPDH levels. Asterisks (*), (**), and (***) indicate statistically significant differences ($p \leqslant 0.05$, $p \leqslant 0.01$, and $p \leqslant 0.001$, respectively) between ORF3a and ORF3a+GSK414. Next to this figure is indicated the corresponding one in the manuscript, in which control and ORF3a group are compared.

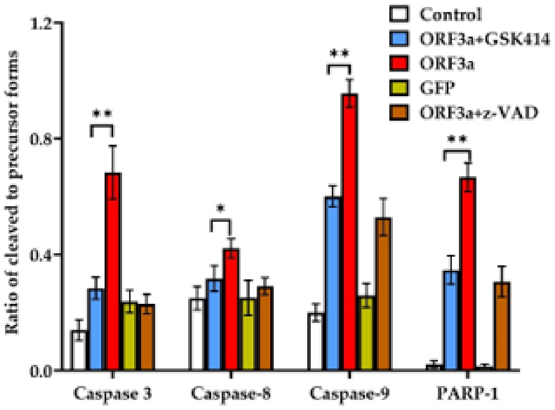

Figure 3e

Figure S7: The relative protein expression levels of caspase -3, -8, -9 (precursor and cleaved forms) and PARP-1 (full length and cleaved form) in protein extracts of control, ORF3a+GSK414, ORF3a, GFP and ORF3a+z-VAD groups. The relative protein expression of all target proteins is normalized to GAPDH protein levels. Asterisks (*), (**), and (***) indicate statistically significant differences ($p \leqslant 0.05$, $p \leqslant 0.01$, and $p \leqslant 0.001$, respectively) between ORF3a and ORF3a+GSK414. Next to this figure is indicated the corresponding one in the manuscript, in which control and ORF3a group are compared.

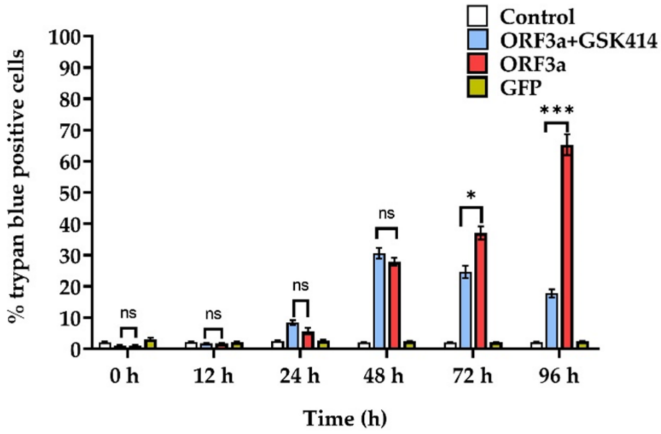

Figure 3h

Figure S8: Evaluation of cell death kinetics during the experimental setup for control, ORF3a+GSK414, ORF3a, GFP using Trypan Blue Dye Exclusion Assay. Measurements were carried out at specific time points 0, 12, 24, 48, 72 and 96 h post transfection. Asterisks (*), (**), and (***) indicate statistically significant differences ($p \leqslant 0.05$, $p \leqslant 0.01$, and $p \leqslant 0.001$, respectively) between ORF3a and ORF3a+GSK414. Next to this figure is indicated the corresponding one in the manuscript, in which control and ORF3a group are compared.

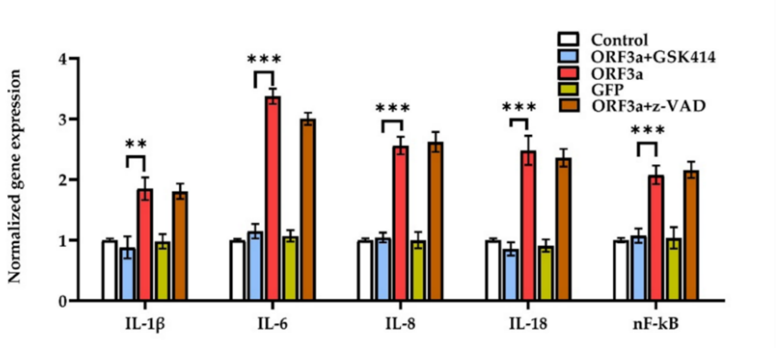

Figure 5a

Figure S9: Assessment of mRNA expression of pro-inflammatory cytokine genes (IL-1b, IL-6, IL-8, IL-18) as well as NFκB in control, ORF3a+GSK414, ORF3a, GFP and ORF3a+z-VAD group at 96 hpt. GAPDH was used as a housekeeping gene. Asterisks (*), (**), and (***) indicate statistically significant differences ($p \leqslant 0.05$, $p \leqslant 0.01$, and $p \leqslant 0.001$, respectively) between ORF3a and ORF3a+GSK414. Next to this figure is indicated the corresponding one in the manuscript, in which control and ORF3a group are compared.

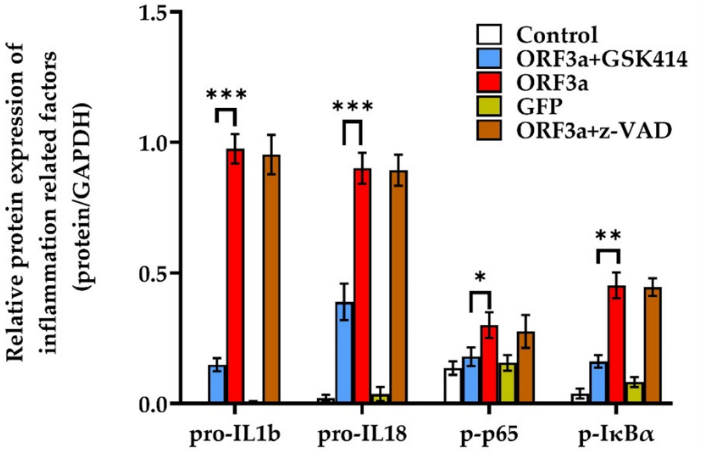

Figure 5c

Figure S10: The relative protein expression levels of IL-1b and IL-18 precursor forms as well as phospho IκB and phospho p65 in cell extracts of control, ORF3a+GSK414, ORF3a, GFP and ORF3a+z-VAD group at 96 hpt The relative protein expression of all inflammatory biomarkers is normalized to GAPDH protein levels. Asterisks (*), (**), and (***) indicate statistically significant differences ($p \leqslant 0.05$, $p \leqslant 0.01$, and $p \leqslant 0.001$, respectively) between ORF3a and ORF3a+GSK414. Next to this figure is indicated the corresponding one in the manuscript, in which control and ORF3a group are compared.
